# *CFTR* variant-associated hereditary pancreatitis complicated by diabetes mellitus: a case report and literature review

**DOI:** 10.3389/fendo.2026.1907178

**Published:** 2026-09-29

**Authors:** Zhenzhen Hong, Xiying Zeng, Jinhui Zhang, Honghong Duan, Yajing Xu

**Affiliations:** 1 Department of Endocrinology, The Second Affiliated Hospital of Fujian Medical University, Quanzhou, Fujian, China; 2 Department of Endocrinology, Huian County General Hospital, Quanzhou, Fujian, China; 3 Department of Obstetrics and Gynecology, The Second Affiliated Hospital of Fujian Medical University, Quanzhou, Fujian, China

**Keywords:** CFTR, familial diabetes, hereditary pancreatitis, pancreatogenic diabetes, type 3C diabetes mellitus

## Abstract

Hereditary pancreatitis (HP) is a rare autosomal dominant disorder characterized by recurrent episodes of pancreatic inflammation leading to chronic pancreatic damage. We report a 38-year-old Chinese male with a heterozygous cystic fibrosis transmembrane conductance regulator (*CFTR*) intronic variant (c.1210-11T>G) who presented with pancreatic diabetes (type 3c diabetes) and an otherwise “silent” pancreatic phenotype. He had a family history of similar manifestations (father and brother with pancreatogenic diabetes). Genetic testing identified the *CFTR* c.1210-11T>G variant, which is known to affect splicing (TG12-5T allele). The variant meets ACMG criteria for pathogenicity. Segregation analysis confirmed all affected relatives carried this variant. No other significant genetic findings (e.g. *SPINK1*, *PRSS1*) were detected. The patient’s presentation was dominated by diabetes with minimal abdominal symptoms; however, pancreatic imaging revealed changes consistent with chronic pancreatitis (pancreatic atrophy and ductal irregularity). The diagnosis of hereditary chronic pancreatitis was established based on clinical, imaging and genetic criteria. In the Discussion, we compare this case to reported *CFTR*-related pancreatitis cases and highlight the importance of considering *CFTR* variants in familial idiopathic pancreatitis with diabetes. This case underscores that *CFTR* single heterozygous variants (especially bicarbonate-defective alleles) can cause a pancreatitis-predominant phenotype. It also emphasizes the need for comprehensive genetic testing in atypical diabetes.

## 1 Introduction

Chronic pancreatitis (CP) is a progressive fibroinflammatory disease of the pancreas characterized by irreversible morphological changes, pancreatic fibrosis, and gradual impairment of both exocrine and endocrine function (1). Pancreatogenic diabetes, also known as type 3c diabetes mellitus (T3cDM), develops secondary to diseases of the exocrine pancreas, with CP being one of the most common underlying causes (2, 3). Unlike type 1 or type 2 diabetes mellitus, T3cDM has distinct etiological, diagnostic, and therapeutic considerations and is frequently under-recognized or misclassified in routine clinical practice, particularly when pancreatic symptoms are mild, absent, or overlooked (4).

Hereditary or genetically determined pancreatitis is most classically associated with gain-of-function variants in PRSS1. However, variants in other pancreatitis-associated genes, including *SPINK1*, *CFTR*, *CTRC* and *CPA1*, have increasingly been implicated in recurrent acute pancreatitis, idiopathic CP, and familial pancreatic disease (5, 6). The cystic fibrosis transmembrane conductance regulator (*CFTR*) gene, originally identified as the causative gene of cystic fibrosis, encodes an epithelial chloride and bicarbonate channel that plays an essential role in maintaining pancreatic ductal fluid secretion (7). Certain *CFTR* variants may predominantly impair pancreatic ductal bicarbonate secretion while sparing sufficient chloride transport in other organs, thereby leading to a pancreas-predominant *CFTR*-related disorder without classic pulmonary manifestations or abnormal sweat chloride findings (8).

In this report, we describe a Chinese male patient with diabetes mellitus and evidence suggestive of *CFTR*-related hereditary CP, in whom genetic testing identified a heterozygous *CFTR* c.1210-11T>G splice-site variant. Segregation analysis further demonstrated the presence of the same variant in affected family members. We review the evidence linking this variant to *CFTR*-related pancreatitis and discuss the diagnostic and clinical implications of *CFTR*-associated pancreatic disease presenting predominantly as familial diabetes mellitus.

## 2 Case presentation

### 2.1 Patient information

A 38-year-old man was referred for genetic evaluation due to early-onset DM. He had been diagnosed with diabetes at age 34 and was managed with oral hypoglycemic agents, achieving reasonable glycemic control. He reported no history of heavy alcohol use or gallstones. The patient denied recurrent abdominal pain or overt acute pancreatitis episodes; however, routine imaging performed for diabetes workup noted pancreatic calcifications and ductal irregularity, raising suspicion for CP. Laboratory tests showed evidence of endocrine pancreatic insufficiency (elevated hemoglobin A1c) but no significant exocrine insufficiency was documented (fecal elastase not tested). Serum amylase and lipase were not markedly elevated at presentation. On physical examination, there was no abdominal tenderness or organomegaly. Body mass index was 24 kg/m^2^. Routine laboratory evaluation was otherwise normal. Given the unusual combination of non-obese diabetes and imaging findings, a pancreatogenic etiology was considered. No sweat chloride testing was performed, as cystic fibrosis was not clinically suspected.

### 2.2 Family history

Detailed family history revealed an apparent autosomal dominant pattern of pancreatic disease. Both the patient’s father (age 60) and paternal uncle had been diagnosed with diabetes (in their 50s) and reportedly experienced episodes of mild acute pancreatitis in middle age. The patient’s younger brother (age 35) also had diabetes and one episode of acute pancreatitis. No family members had classic respiratory manifestations of CF. These findings raised suspicion for an inherited cause of pancreatitis/diabetes, prompting comprehensive genetic testing for hereditary pancreatitis and diabetes genes.

### 2.3 Genetic testing results

Genomic DNA extracted from peripheral blood of the proband was analyzed using a targeted next-generation sequencing panel covering 166 genes associated with hereditary diabetes, including genes related to maturity-onset diabetes of the young, neonatal diabetes, and hereditary pancreatitis. Sequencing analysis identified a heterozygous splice-region variant in the *CFTR* gene: NM_000492.4.1210-11T>G, located in intron 9. According to the American College of Medical Genetics and Genomics and Association for Molecular Pathology (ACMG/AMP) criteria, this variant was classified as pathogenic.

The evidence supporting pathogenicity included the following: PVS1_Strong, as previous RT-PCR functional studies have demonstrated that this intronic variant causes abnormal mRNA splicing and exon 10 skipping, resulting in markedly impaired *CFTR* transcript/protein function (9); PM1, as this variant is located in a functionally important *CFTR* splice-regulatory region and has been recognized as one of the pathogenic *CFTR* variants associated with *CFTR*-related disorders and CP (10); and PM3_Very Strong, as this variant has been reported in multiple patients with *CFTR*-related disease in trans with other pathogenic *CFTR* variants, including c.[350G>A];[1210-11T>G], c.[1521_1523del];[1210-11T>G], c.[1364C>A];[1210-11T>G], and c.[695T>A];[1210-11T>G] (11). In addition, this variant is extremely rare in population databases, with the highest reported population frequency of approximately 0.024% in ESP, 1000 Genomes, and gnomAD, further supporting its pathogenic relevance.

A second heterozygous variant of uncertain significance was detected in the *HADH* gene, NM_005327.7.863A>C (p.Gln288Pro). Given the autosomal recessive inheritance pattern of HADH-related disorders and the heterozygous status of this variant in the proband, it was not considered the primary genetic cause of the patient’s phenotype.

### 2.4 Segregation analysis

To assess co-segregation, targeted Sanger sequencing of the *CFTR* region was performed using genomic DNA extracted from peripheral blood samples of available family members. The proband, his father, and his diabetic brother were confirmed to carry the same heterozygous *CFTR* variant, NM_000492.4.1210-11T>G (**Figure 1**). These findings suggest limited co-segregation between the *CFTR* variant and the familial diabetes phenotype among the tested individuals. However, because pancreatitis-related phenotypes were incompletely documented and the number of genetically tested relatives was limited, this result should not be interpreted as definitive evidence of autosomal dominant hereditary pancreatitis in this family.

**Figure 1:**
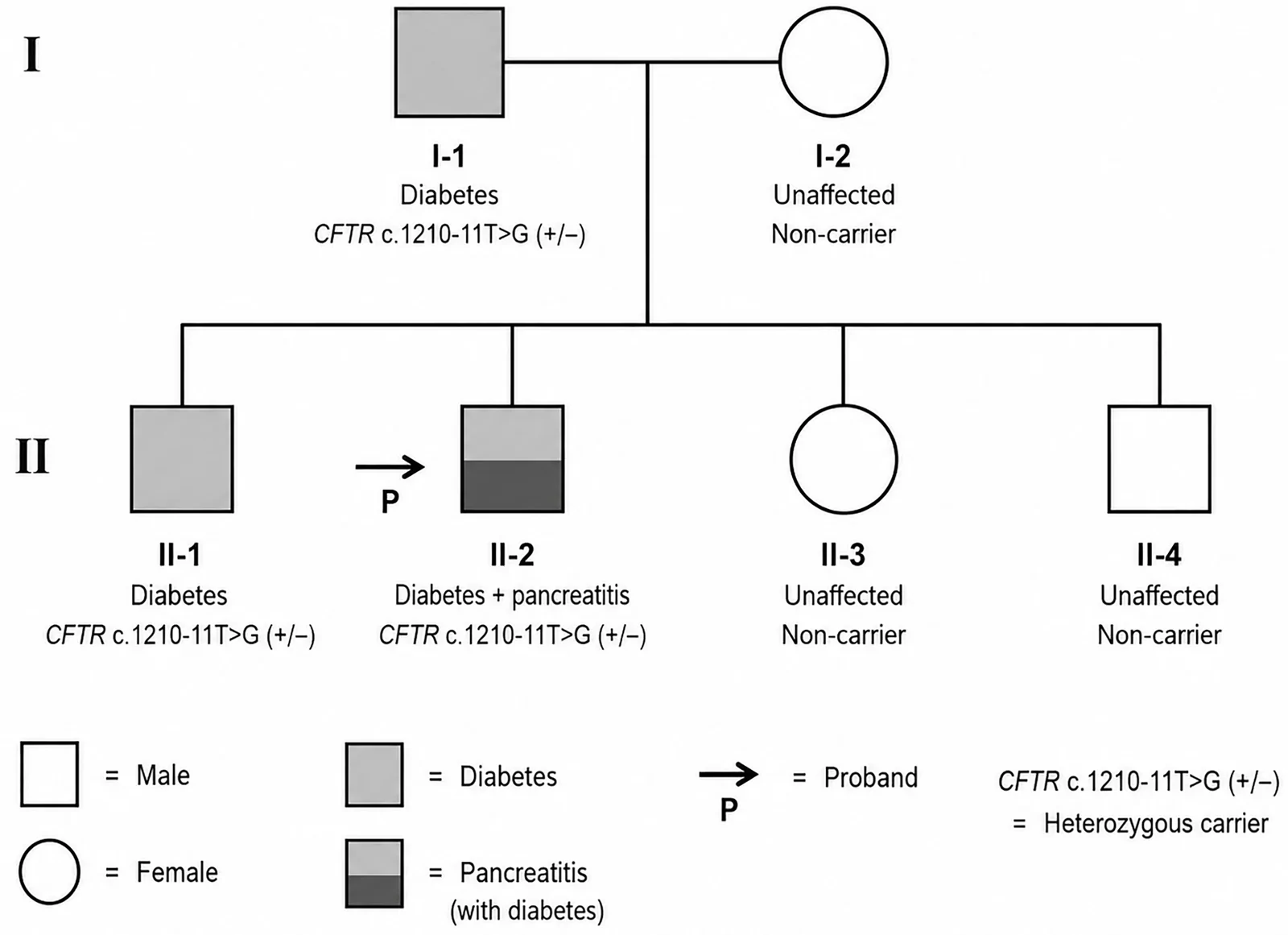
Pedigree of the family showing diabetes/pancreatitis status and segregation of the heterozygous CFTR c.1210-11T>G variant. Squares and circles represent male and female family members, respectively. The proband (II-2) is indicated by an arrow and the letter “P”. Light gray shading denotes diabetes mellitus, and the dark lower segment denotes pancreatitis. Genotyping results for CFTR NM_000492.4:c.1210-11T>G are shown below each individual; “+/−” indicates heterozygosity, and “non-carrier” indicates that the variant was not detected. Siblings are arranged from left to right according to birth order.

### 2.5 Sanger sequencing verification

Sanger sequencing confirmed the presence of the c.1210-11T>G variant in the proband (**Figure 2**). The chromatogram shows overlapping peaks (G/T) at the splice donor site, consistent with a heterozygous change. The same heterozygous sequence was observed in affected family members, and was absent in unaffected members, confirming the NGS result.

**Figure 2:**
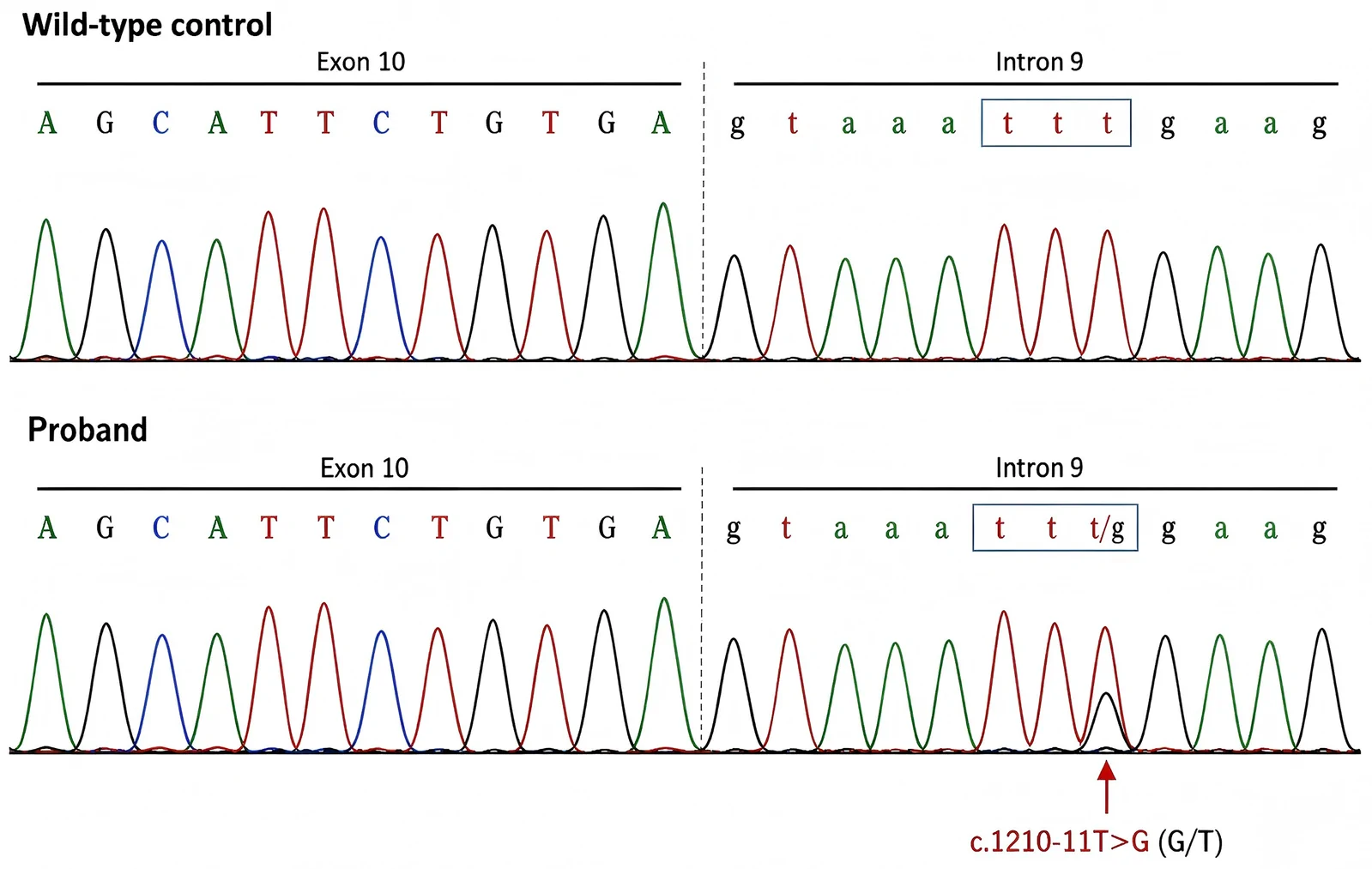
Partial Sanger sequencing chromatograms of CFTR exon 10/intron 9 junction. Top: wild-type control sequence (TTT variant at intron 9). Bottom: proband sequence showing heterozygosity for the c.1210-11T>G variant (arrow, red peak on G>T change).

### 2.6 Post-diagnosis management and follow-up

After the identification of the heterozygous *CFTR* splice-region variant, no *CFTR* modulator therapy was initiated. Although the variant was classified as pathogenic by the testing laboratory, the patient did not have a confirmed clinical diagnosis of cystic fibrosis, and the available materials did not provide objective evidence of chronic pancreatitis, exocrine pancreatic insufficiency, or a *CFTR* modulator-responsive clinical phenotype. Therefore, the genetic finding was considered insufficient to justify targeted *CFTR*-directed therapy at this stage.

The post-diagnosis management strategy focused on continued diabetes care, recommendation for further pancreatic phenotyping, and genetic counseling. Further evaluation with pancreatic imaging, serum pancreatic enzyme levels, fecal elastase-1, C-peptide measurement, and diabetes-associated autoantibodies was recommended to better define the diabetes subtype and assess whether an occult *CFTR*-related pancreatic phenotype was present. Available source materials did not include a detailed longitudinal follow-up period or objective glycemic response after these recommendations. This lack of follow-up data was considered an important limitation of the present report.

## 3 Diagnosis

Based on the constellation of findings—familial history, imaging evidence of CP (ductal irregularity, calcifications), absence of other causes (no alcohol abuse, normal triglycerides), and a pathogenic *CFTR* variant—we diagnose *CFTR*-related hereditary CP with secondary diabetes (pancreatic endocrine insufficiency). Acute pancreatitis (AP) is classically diagnosed when ≥2 of: (1) acute onset of epigastric pain, (2) serum amylase or lipase ≥3× normal, (3) consistent imaging findings. In this patient, documented episodes may have been mild or subclinical, but the imaging (CT/MR) findings satisfied criteria for CP (pancreatic atrophy, calcifications) in the context of genetic predisposition. The diabetes is attributed to chronic pancreatic dysfunction (Type 3c DM).

The *CFTR* c.1210-11T>G variant (TG12-5T) is known to reduce *CFTR* function by aberrant splicing. It has been previously reported in *CFTR*-related disorders (e.g., congenital bilateral absence of vas deferens) and pancreatitis cohorts. According to ACMG guidelines and *CFTR* expert recommendations, this variant meets criteria for pathogenicity. Notably, similar *CFTR* variants that predominantly impair bicarbonate secretion (but spare chloride) cause pancreatic disease without typical cystic fibrosis lung findings. Thus, a single heterozygous TG12-5T change can be sufficient for an autosomal dominant pancreatitis-predominant phenotype (OMIM 167800). We conclude that this *CFTR* mutation is the primary genetic etiology of the patient’s pancreatopathy.

## 4 Discussion

### 4.1 *CFTR* c.1210-11T>G pathogenicity and pancreas-predominant disease

The c.1210-11T>G variant is located in the intron 9 poly-T/TG tract region of *CFTR* and is often discussed in relation to the TG12T5 allele (12). This region is known to influence exon 10 splicing, and functional studies have suggested that TG12T5 can reduce normal *CFTR* mRNA and protein expression (13). Available *in vitro* and clinical data support the interpretation of this allele as clinically relevant, particularly when it occurs in trans with another pathogenic *CFTR* variant, whereas its effect in the heterozygous state may be variable and context-dependent (9).

To further clarify the clinical implication of this variant, we reviewed the available *CFTR2*-related information. According to *CFTR2*-based interpretation, TG12T5 is better regarded as a variant of varying clinical consequence rather than a uniformly classic CF-causing variant. Its phenotypic effect depends on the adjacent TG repeat number, the variant present on the other *CFTR* allele, and the clinical phenotype. Therefore, in the present case, the heterozygous *CFTR* c.1210-11T>G finding should be interpreted as a possible contributor to a *CFTR*-related pancreatic phenotype rather than as definitive proof of autosomal dominant hereditary pancreatitis.

Importantly, the net effect of a *CFTR* genotype may be influenced by the functional severity of each allele and by organ-specific vulnerability. Certain bicarbonate-defective variants have been associated with pancreatitis-predominant phenotypes, in which pancreatic ductal bicarbonate secretion is impaired while pulmonary manifestations may be absent or mild. In this context, the *CFTR* c.1210-11T>G variant may raise suspicion of an occult *CFTR*-related pancreatic phenotype. However, because the present patient carried this variant in the heterozygous state and lacked complete pancreatic phenotyping, including pancreatic imaging and exocrine function testing, the causal relationship between this variant and the diabetes-dominant presentation remains uncertain. The segregation data support only limited co-segregation with familial diabetes, rather than definitive segregation with hereditary pancreatitis.

### 4.2 *CFTR* and familial idiopathic diabetes

This case illustrates that a possible *CFTR*-related pancreatic phenotype should be considered in selected patients initially classified as having type 2 diabetes, particularly when familial young-onset diabetes and an atypical clinical profile are present. Previous studies have reported that some patients with apparently idiopathic diabetes or pancreatogenic diabetes may later be found to have pancreatitis-associated gene variants, including *CFTR* or *PRSS1* variants. In the present case, the strong family history and young-adult onset of diabetes prompted consideration of a possible inherited or pancreatogenic contribution, although definitive classification was limited by incomplete metabolic and pancreatic phenotyping. Pancreatic diabetes (T3cDM) is under-recognized; one review notes that many patients with CP carry modulating gene variants and are often misdiagnosed with type 2 DM (14). Early recognition of a possible genetic contribution to pancreatic or diabetes phenotypes is important because it may guide further pancreatic evaluation, lifestyle counseling, genetic counseling, and individualized follow-up (15).

### 4.3 Additional interpretation of the *HADH* variant

The additional *HADH* variant, NM_005327.7.863A>C (p.Gln288Pro), was classified as a variant of uncertain significance. *HADH* encodes hydroxyacyl-CoA dehydrogenase and is classically associated with autosomal recessive metabolic disorders, particularly congenital hyperinsulinemic hypoglycemia and fatty acid oxidation-related phenotypes. In this case, the patient carried a heterozygous *HADH* variant, and the available materials did not document congenital hyperinsulinism, recurrent hypoglycemia, abnormal acylcarnitine profile, or other features suggestive of a fatty acid oxidation disorder. Therefore, this variant was considered insufficient to explain the familial young-onset diabetes phenotype. However, because no functional validation or extended segregation analysis was available, a potential modifier role cannot be completely excluded and remains speculative.

### 4.4 Literature on *CFTR* variants and pancreatitis

*CFTR* variants have been increasingly recognized in CP cases. For example, heterozygous *CFTR* mutations are found in up to 43% of idiopathic recurrent acute pancreatitis and 11% of CP patients. Known pancreatitis-associated variants include mild classes (e.g. R75Q, R117H, 5T alleles) often in trans with other alleles. A recent case series (Yıldız 2025) reported a 74-year-old male with recurrent pancreatitis harboring heterozygous c.3154T>G (p.Phe1052Val), supporting a possible association with hereditary or genetic pancreatitis in that case. Representative examples have been summarized in the literature. Unlike those cases, our patient had c.1210-11T>G alone in the heterozygous state. This rare intronic variant has also been reported in a Japanese child with cystic fibrosis (compound homozygote).To our knowledge, reports describing TG12T5 in Asian families with familial young-onset diabetes and suspected pancreatic involvement remain limited.

### 4.5 Clinical implications

This case has several implications for clinical practice. First, it underscores the need to consider pancreatitis-associated genes, including *CFTR*, in selected patients with familial young-onset diabetes, especially when the diabetes phenotype is atypical or when there is suspicion of occult pancreatic involvement. Standard MODY panels or neonatal diabetes gene panels may not include *CFTR*; therefore, broad next-generation sequencing panels or targeted *CFTR* analysis may be informative when clinical suspicion is high (16).

Second, the identification of a pathogenic *CFTR* variant should prompt phenotype-driven assessment rather than immediate diagnostic closure. In the present case, the genetic result raised the possibility of an unrecognized *CFTR*-related pancreatic phenotype, but it was not sufficient to establish a definitive diagnosis of hereditary pancreatitis or type 3c diabetes. Further evaluation with pancreatic imaging, serum pancreatic enzyme levels, fecal elastase-1, C-peptide measurement, and diabetes-associated autoantibodies would be useful to clarify the diabetes subtype and assess possible pancreatic involvement.

Third, from a therapeutic perspective, the identification of a pathogenic *CFTR* variant does not automatically indicate eligibility for *CFTR* modulator therapy. Current *CFTR* modulators are primarily used in patients with a clinical diagnosis of cystic fibrosis who carry *CFTR* variants known to be responsive to these agents. In the present case, the patient had a heterozygous *CFTR* splice-region variant but no confirmed diagnosis of cystic fibrosis, no documented chronic pancreatitis, and no objective evidence of exocrine pancreatic insufficiency in the available records. Therefore, *CFTR* modulator therapy was not initiated. Instead, the most appropriate management approach was considered to be phenotype-driven follow-up, including structured pancreatic assessment, continued diabetes management, avoidance of pancreatic toxins, and genetic counseling.

Finally, awareness that *CFTR* variants may be associated with pancreatic phenotypes can help endocrinologists consider occult pancreatic disease in selected patients presenting primarily with endocrine manifestations (17). If future evaluation confirms clinically significant *CFTR*-related pancreatic disease or chronic pancreatitis, management may include pancreatic enzyme replacement when exocrine pancreatic insufficiency is present, individualized glycemic management, lifestyle modification, and specialist assessment of long-term pancreatic cancer risk.

## 5 Conclusion

We report a rare case of heterozygous *CFTR* c.1210-11T>G (TG12T5) variant in a Chinese patient presenting with pancreatogenic diabetes. Despite lack of classic cystic fibrosis findings, this mutation likely caused a subclinical CP leading to diabetes. This highlights that *CFTR*-related pancreatitis can manifest predominantly as DM and may be underdiagnosed. Genetic testing provided definitive diagnosis, guiding appropriate management. We recommend that clinicians consider *CFTR* genetic analysis in patients with idiopathic familial diabetes or pancreatitis, and that *CFTR* variants affecting bicarbonate conductance (TG12T5, R75Q, etc.) be recognized as possible causes of pancreatitis-predominant disease.

## Data Availability

The original contributions presented in the study are included in the article. Further inquiries can be directed to the corresponding author.

## References

[1] Cohen SM Kent TS . Etiology, diagnosis, and modern management of chronic pancreatitis: A systematic review. JAMA Surg. (2023) 158:652–61. doi: 10.1001/jamasurg.2023.0367 37074693

[2] Rasheed A Galande S Farheen S Mitnala S Reddy DN Talukdar R . Type 3c diabetes associated with chronic pancreatitis: A narrative review. Pancreatology Off J Int Assoc Pancreatology (IAP). (2025) 25:1003–12. doi: 10.1016/j.pan.2025.08.005 40819996

[3] Hart PA Bellin MD Andersen DK Bradley D Cruz-Monserrate Z Forsmark CE . Type 3c (pancreatogenic) diabetes mellitus secondary to chronic pancreatitis and pancreatic cancer. Lancet Gastroenterol Hepatol. (2016) 1:226–37. doi: 10.1016/s2468-1253(16)30106-6 28404095 PMC5495015

[4] Hardt PD Brendel MD Kloer HU Bretzel RG . Is pancreatic diabetes (type 3c diabetes) underdiagnosed and misdiagnosed? Diabetes Care. (2008) 31:S165–9. doi: 10.2337/dc08-s244 18227480

[5] Rosendahl J Landt O Bernadova J Kovacs P Teich N Bödeker H . CFTR, SPINK1, CTRC and PRSS1 variants in chronic pancreatitis: Is the role of mutated CFTR overestimated? Gut. (2013) 62:582–92. doi: 10.1136/gutjnl-2011-300645 22427236

[6] Zou WB Tang XY Zhou DZ Qian YY Hu LH Yu FF . SPINK1, PRSS1, CTRC, and CFTR genotypes influence disease onset and clinical outcomes in chronic pancreatitis. Clin Trans Gastroenterol. (2018) 9:204. doi: 10.1038/s41424-018-0069-5 30420730 PMC6232107

[7] Jiang J Waidyaratne G Mussad S Harris S Roberts ME Gokun Y . Prevalence of CFTR pathogenic variants in pancreatitis: A systematic review and meta-analysis. Clin Trans Gastroenterol. (2025) 16:e00846. doi: 10.14309/ctg.0000000000000846 40249094 PMC12330363

[8] Berke G Gede N Szadai L Ocskay K Hegyi P Sahin-Tóth M . Bicarbonate defective CFTR variants increase risk for chronic pancreatitis: A meta-analysis. PloS One. (2022) 17:e0276397. doi: 10.1371/journal.pone.0276397 36264955 PMC9584382

[9] Delaney SJ Rich DP Thomson SA Hargrave MR Lovelock PK Welsh MJ . Cystic fibrosis transmembrane conductance regulator splice variants are not conserved and fail to produce chloride channels. Nat Genet. (1993) 4:426–31. doi: 10.1038/ng0893-426 7691356

[10] Steiner B Rosendahl J Witt H Teich N Keim V Schulz HU . Common CFTR haplotypes and susceptibility to chronic pancreatitis and congenital bilateral absence of the vas deferens. Hum Mutat. (2011) 32:912–20. doi: 10.1002/9781118997505.ch49 21520337

[11] Niksic M Romano M Buratti E Pagani F Baralle FE . Functional analysis of cis-acting elements regulating the alternative splicing of human CFTR exon 9. Hum Mol Genet. (1999) 8:2339–49. doi: 10.1093/hmg/8.13.2339 10556281

[12] LaRusch J Jung J General IJ Lewis MD Park HW Brand RE . Mechanisms of CFTR functional variants that impair regulated bicarbonate permeation and increase risk for pancreatitis but not for cystic fibrosis. PloS Genet. (2014) 10:e1004376. doi: 10.1371/journal.pgen.1004376 25033378 PMC4102440

[13] Bombieri C Claustres M De Boeck K Derichs N Dodge J Girodon E . Recommendations for the classification of diseases as CFTR-related disorders. J Cystic Fibrosis Off J Eur Cystic Fibrosis Soc. (2011) 10:S86–S102. doi: 10.1016/s1569-1993(11)60014-3 21658649

[14] Rickels MR Bellin M Toledo FGS Robertson RP Andersen DK Chari ST . Detection, evaluation and treatment of diabetes mellitus in chronic pancreatitis: Recommendations from PancreasFest 2012. Pancreatology Off J Int Assoc Pancreatology (IAP). (2013) 13:336–42. doi: 10.1016/j.pan.2013.05.002 23890130 PMC3830751

[15] Ode KL Imai Y Norris AW . Approach to the patient with pancreatogenic diabetes. J Clin Endocrinol Metab. (2026) 111:e569–76. doi: 10.1210/clinem/dgaf623 41252284 PMC12819855

[16] Weiss FU . Pancreatic cancer risk in hereditary pancreatitis. Front Physiol. (2014) 5:70. doi: 10.3389/fphys.2014.00070 24600409 PMC3929831

[17] Kim HS Gweon TG Park SH Kim TH Kim CW Chang JH . Incidence and risk of pancreatic cancer in patients with chronic pancreatitis: Defining the optimal subgroup for surveillance. Sci Rep. (2023) 13:106. doi: 10.1038/s41598-022-26411-8 36596818 PMC9810784

